# Two cases of spindle cell tumors with S100 and CD34 co-expression showing novel *RAF1* fusions

**DOI:** 10.1186/s13000-022-01263-y

**Published:** 2022-10-13

**Authors:** Li-Hua Gong, Wei-Feng Liu, Xiao-Hui Niu, Yi Ding

**Affiliations:** 1 Department of Pathology, Beijing Jishuitan Hospital, The Fourth Medical College of Peking University, 100035 Beijing, China; 2 Department of orthopedic oncology, Beijing Jishuitan Hospital, The Fourth Medical College of Peking University, 100035 Beijing, China

**Keywords:** RAF, S100 and CD34 co-expression, Soft tissue tumors

## Abstract

**Background:**

Recently, a novel group of CD34 and S100 co-expression spindle cell tumors with distinctive stromal and perivascular hyalinization harboring recurrent gene fusions involving *RET*, *RAF1*, *BRAF*, and *NTRK1/2* gene has been identified.

**Case presentation:**

In this study, we reported two Chinese male patients with soft tissue tumors presenting in the right knee joint and the left thigh, respectively. For both patients, the tumors were completely excised with clear margin. Microscopically, case 1showed morphological overlap with neurofibroma, and case 2 showed overlap with lipomatous solitary fibrous tumor. Both tumors showed co-expression of S100 and CD34, and absence of SOX10. Genomic profiling with DNA-based next-generation sequencing (NGS) assay was performed and revealed *KIF5B-RAF1* (K16:R8) and *TLN2-RAF1* (T54:R8) rearrangements. RNA-based NGS and RT-PCR were performed to confirm the gene fusion.

**Conclusions:**

Though systemic therapy was not indicated in these two patients, identification of targetable kinase fusions may help to refine tumors with an ambiguous immunoprofile, and provides suggestions for targeted therapy in rare aggressive cases.

**Supplementary Information:**

The online version contains supplementary material available at 10.1186/s13000-022-01263-y.

## Background

Recently, a series of low to intermediate grades of soft tissue tumors showing recurrent fusions involving *RAF1*, *BRAF*, and *NTRK1/2* genes have been defined as a new entity with S100 and CD34 co-expression (without SOX-10 positivity) (1). These tumors exhibit a spectrum of histological features, including monomorphic spindle cell proliferation, “patternless” growth pattern, stromal and perivascular hyalinization. Herein, we report two morphologically distinct, S100 and CD34 co-expressed low-grade soft tissue tumors carrying novel *RAF1* gene fusions with *kinesin family member 5B* (*KIF5B*) and *talin2* (*TLN2*), respectively.

## Case presentation

### Case 1

was a 38-year-old male who presented with an asymptomatic, painless mass in his right knee joint. Ultrasound revealed a 7.7 × 6.2 × 2.5 cm irregular hypoechoic mass, with unclear boundary and abundant blood flow signals inside. Magnetic resonance imaging (MRI) confirmed a lobulated intramuscular mass in his right thigh. Case 2 was a 15-year-old boy who complained of an asymptomatic, painless mass in his left thigh. For both patients, the tumors were completely excised with a clear margin. Subsequent 9-month follow-up of case 1and 6-month follow-up of case 2 did not reveal local or distant recurrence by clinical examination and MRI.

**Fig. 1:**
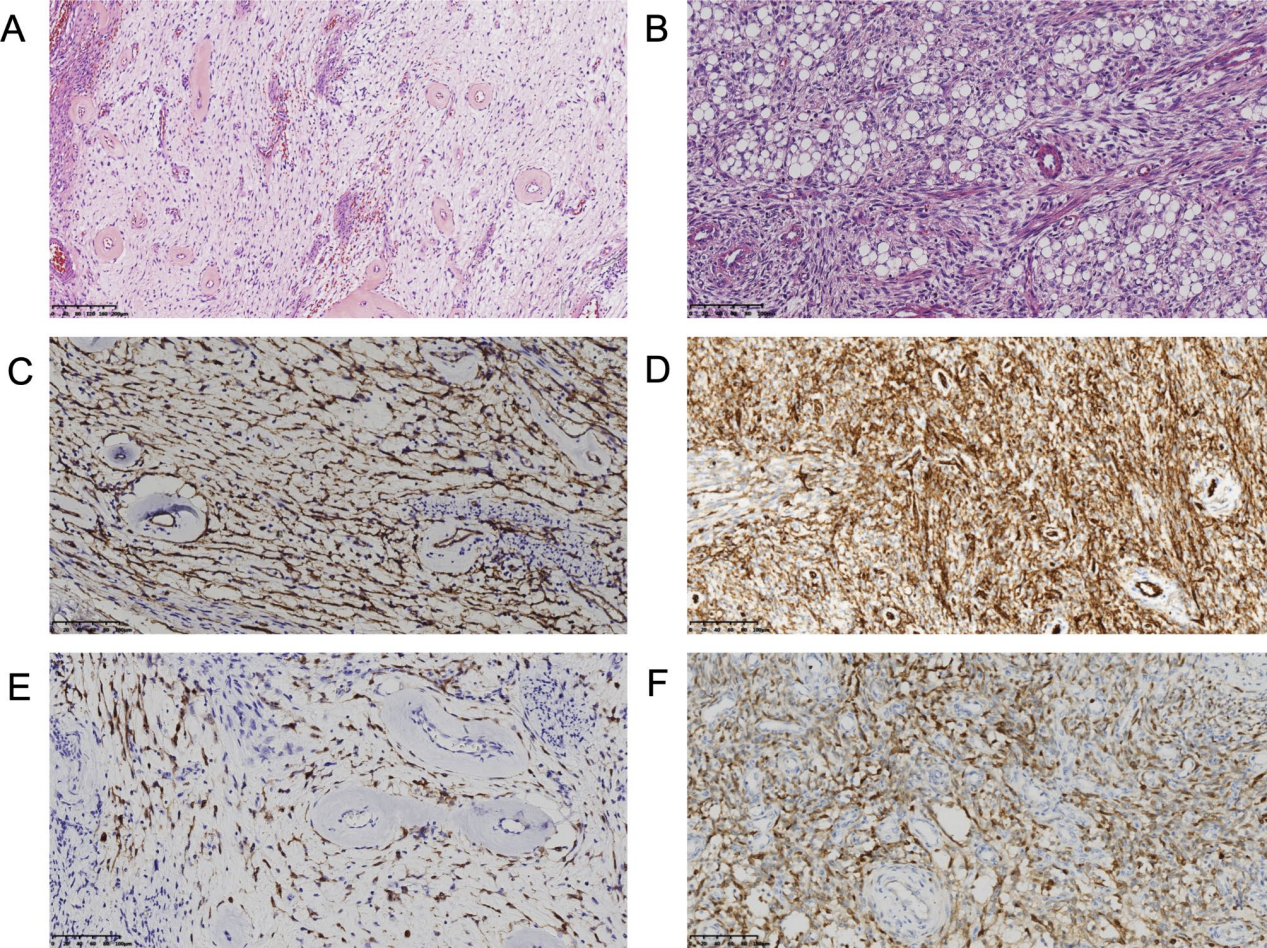
Histologic and immunohistochemical features of *RAF1*-rearranged tumors. A, Neurofibroma like tumor in a 30-year-old male showing slatternly arranged short spindle cells with striking perivascular rings of collagen (case1 ; ×20). B, LSFT -like tumor in a 15-year-old male showing diffusely arranged spindle cells without obvious atypia and mitotic figures (case 2; ×20). C, E, Immunohistochemical studies showing diffuse S100 and CD34 positivity (case1 ; ×20). D, F, Immunohistochemically, the tumor cells showed diffuse expression of S100 and CD34 (case 2; ×20)

Among soft tissue tumors with CD34 and S100 expression, neurofibroma and malignant peripheral nerve sheath tumor (MPNST) are the main differential diagnosis. In case1 , mucinous degeneration was obvious. Short spindle cells were scattered. The tumor showed the morphological characteristic of neurofibroma (Fig. 1 A). In case 2, the spindle cells proliferated diffusely with no obvious atypia. Mitotic figures were rare. The tumor was composed of mature adipocytes admixed with spindle cells, showing lipomatous solitary fibrous tumor (LSFT) like morphology (Fig. 1 B). Immunohistochemically, both cases shared the same profile. The tumors showed diffuse and strong expression of S100 protein (Fig. 1 C,D), CD34 (Fig. 1E F), and absence of SOX10 positivity. The cells also showed positive staining for vimentin, H3K27Me3, CD31, and SMA. Immunostains were negative for pan-TRK, CK, EMA, ERG, CD68, Desmin, MyoD1, Myogenin, TLE1 and STAT6. Furthermore, Ki67 index of the patient 1 and patient 2 samples were 20% and 10%, respectively. We considered that both patients had a low-grade soft tissue tumor with S100 and CD34 co-expression.

**Fig. 2:**
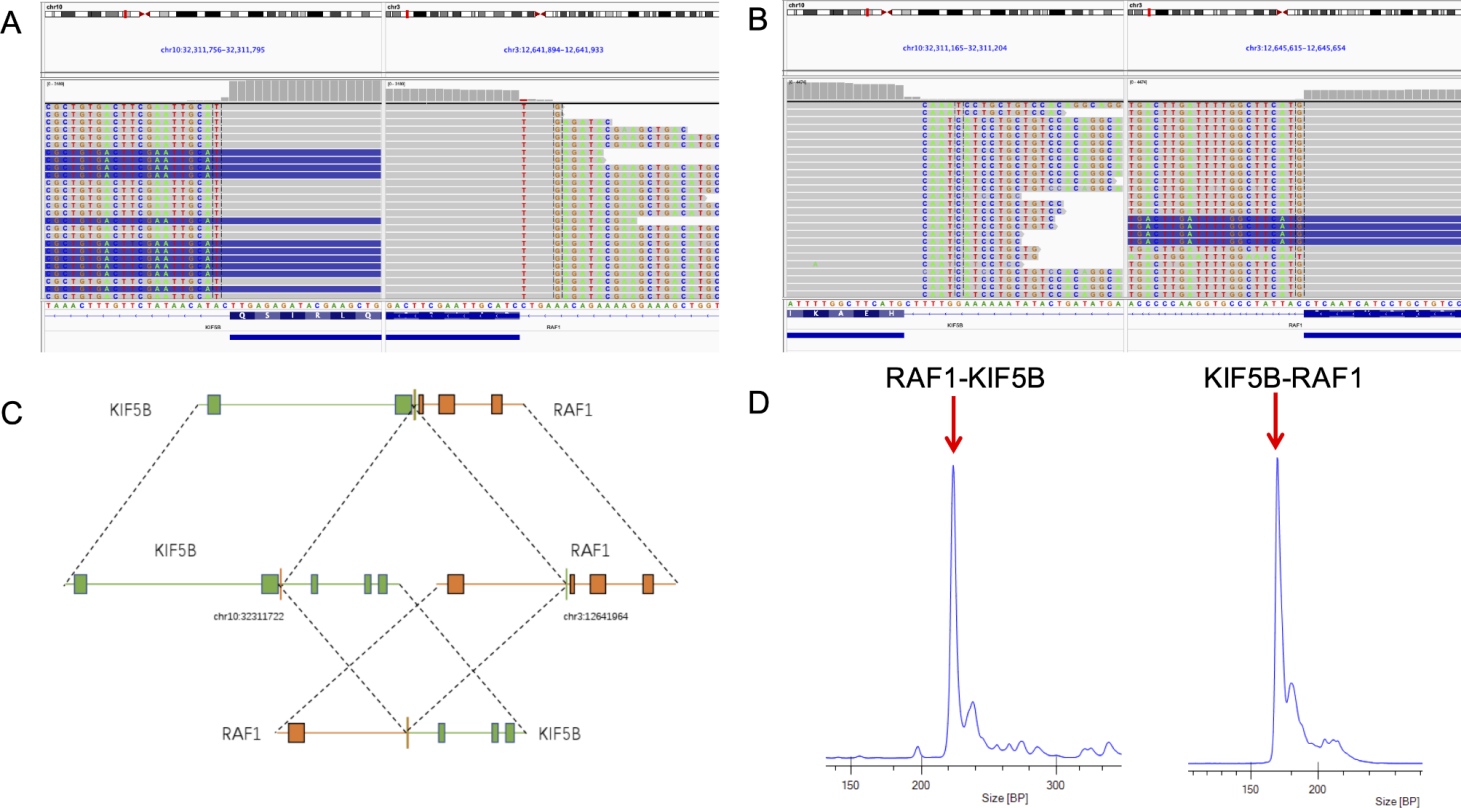
Genomic structures of the *KIF5B-RAF1* and *RAF1-KIF5B* fusions identified in tumor are shown based on RNA-based NGS. A, B, Breakpoints were detected in intron 8 of *KIF5B* and intron 3 of *RAF1*, separately. The predicated resultant gene fusions were between exon 16 of *KIF5B* and exon 8 of *RAF1*, and exon 7 of *RAF-1* and exon 17 of *KIF5B*. C. Schematic representation of the *KIF5B-RAF1* and *RAF1-KIF5B* fusion transcripts. D, RT-PCR amplicon product revealed the *KIF5B -RAF1* and *RAF1-KIF5B* fusion transcripts were at right size (170 bp, 221 bp; red arrows)

**Fig. 3:**
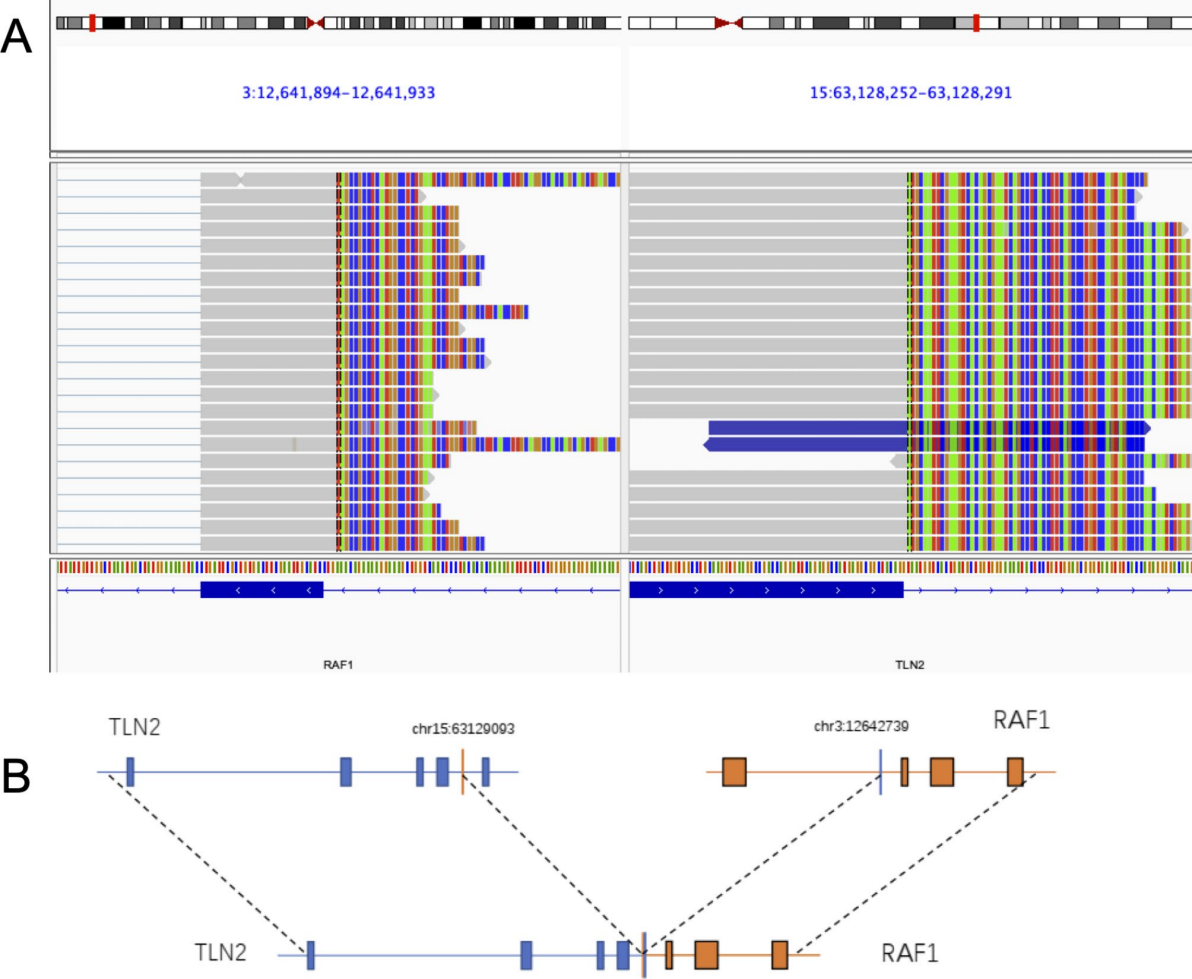
Genomic structure of the *TLN2-RAF1* fusion identified in tumor is shown based on RNA-based NGS. A, Breakpoints were detected in intron 54 of *TLN2* and intron 7 of *RAF1*, separately. The predicated resultant gene fusion was between exon 54 of *TLN2* and exon 8 of *RAF1*. C. Schematic representation of the *TLN2-RAF1* fusion transcript

Given the uncertainty in diagnosis, genomic alternations were studied with both DNA-based next-generation sequencing (NGS) assay (Burning Rock OncoScreen Plus ®, Burning Rock Biotech, Guangzhou, China) and targeted RNA platform (OncoRNA, Burning Rock Biotech, Guangzhou, China) to identify additional molecular alterations and to potentially aid in patient management. DNA- and RNA-based NGS identified gene fusions of *RAF1* exon 8 to *KIF5B* exon 16 in case1 , and RAF1 exon 8 to T*LN2* exon 54 in case 2. In case1 , DNA-based NGS identified a *KIF5B-RAF1* (K16:R8) rearrangement resulting from an in frame t(10;3) (q21.3; q11.2) translocation. RNA-based NGS confirmed these uncommon *RAF1* rearrangements from DNA sequencing, and additionally identified a reciprocal *RAF1- KIF5B* (R7:K17) fusion transcript (Fig. 2 A-C). The *KIF5B-RAF1* and *RAF1- KIF5B* fusions were further confirmed by reverse-transcriptase polymerase chain reaction (RT-PCR; Fig. 2D). In case 2, DNA-based NGS identified a *TLN2-RAF1* (T54:R8) rearrangement resulting from an in-frame t(15;3) (q21.1; q11.2) translocation, which was confirmed by RNA-based NGS (Fig. 3 A, B). Based on the clinicopathologic and genomic features of the two cases, *RAF 1* fusion-positive soft tissue tumor with S100 and CD34 co-expression was the final diagnosis.

## Discussion and conclusions

RAF1 is a member of the RAF family of signaling kinases, downstream of RAS, which activates the MEK/ERK pathway that promotes cell proliferation and survival. In soft tissue tumors with S100 and CD34 co-expression, at least 4 *RAF1* fusion partner genes have been identified to date, including *PDZRN3*, *SLMAP*, and *TMF1* genes located on chromosome 3 and *MTAP* gene located on chromosome 9 (1–3). *TLN2* on chromosome 15 and *KIF5B* on chromosome 10 are first identified as *RAF1* fusion partners in our study. Both *KIF5B-RAF1* and *TLN2-RAF1* fusion transcripts conserve the RAF1 tyrosine kinase domain with the loss of the autoinhibitory region upstream. *KIF5B* and *TLN2* genes retained their promoters to initiate transcription, which then leads to the activation of *RAF1* and the downstream MAPK/ERK pathway. *KIF5B* has been reported as a partner gene in lung adenocarcinomas (LADCs), typically in relation to pericentric inversion events of the *RET* proto-oncogene (4). *TLN2* has been rarely reported as a partner gene in tumors.

Our cases revealed the genetic diversity of *RAF1* fusions, and also underscored the need for diagnostic strategies that are fusion partner non-specific. Common methods for detecting gene fusions in the clinic include RT-PCR, break-apart fluorescence in situ hybridization (FISH), and DNA/RNA-based NGS assay. The main advantage of RT-PCR lies in the possibility of identifying specific known *RAF1* fusion partners. The performance of RT-PCR is limited by atypical *RAF1* rearrangement with new fusion partners and RNA quality. Break-apart FISH plays a critical role in fusion screening tests given its single-cell resolution and rapid turn-around time. Positive FISH results with unusual, complex rearranged patterns and borderline-negative FISH results may benefit from further confirmation with RNA-sequencing. DNA-based NGS is preferable to FISH as a screen due to higher sensitivity for all gene fusions including exons and introns near the kinase domain. However, any atypical *RAF1* rearrangements with new fusion partners should be further evaluated by RNA sequencing to confirm the presence of the fusion transcript. The utility of RNA sequencing for confirming unusual rearrangements from DNA sequencing or break-apart FISH could improve the quality and accuracy of *RAF1* fusion testing.

In conclusion, we reported two cases of *RAF1* fusion transcripts in soft tissue tumor with CD34 and S100 co-expression, extending the molecular genetic spectrum of this recently described entity. Though systemic therapy was not indicated in these two patients, identifying targetable kinase fusions involving the MAPK/ERK pathway may help refine tumors with an ambiguous immunoprofile, and provide new paths for precision medicine strategies involving targeted kinase inhibitors.

## Electronic supplementary material

Below is the link to the electronic supplementary material.

Supplementary Material 1

## Data Availability

The datasets used and/or analysed during the current study are available from the corresponding author on reasonable request.

## List of abbreviations

NGS: next-generation sequencing.
MRI: Magnetic resonance imaging.
