# Supplementary material for: Two cases of spindle cell tumors with S100 and CD34 co-expression showing novel RAF1 fusions
Source: Diagn Pathol. 2022 Oct 13;17:80. doi: 10.1186/s13000-022-01263-y (PMC9563504; doi:10.1186/s13000-022-01263-y)
Supplement: Supplementary file 1 — Supplementary Material 1 [file 13000_2022_1263_MOESM1_ESM.docx]

**Supplementary table 1:** The primer sequences of *KIF5B*-*RAF1* were used in RT-PCR

| The primer sequences |  |
| --- | --- |
| Forward primer | 5' TCATCTGAAGGTTCCCTCTCCC 3' |
| Reverse primer | 5' TTCTCTTGTGCTCGAAGCTGGA 3' |
